# Quantitative vs. Qualitative SPECT-CT Diagnostic Accuracy in Bone Lesion Evaluation—A Review of the Literature

**DOI:** 10.3390/diagnostics13182971

**Published:** 2023-09-17

**Authors:** Mario-Demian Mutuleanu, Diana Loreta Paun, Alexandra Maria Lazar, Cristina Petroiu, Oana Gabriela Trifanescu, Rodica Maricela Anghel, Mirela Gherghe

**Affiliations:** 1 Nuclear Medicine Department, University of Medicine and Pharmacy “Carol Davila”, 050474 Bucharest, Romania; 2 Nuclear Medicine Department, Institute of Oncology “Prof. Dr. Alexandru Trestioreanu”, 022328 Bucharest, Romania; alexandra-maria.lazar@rez.umfcd.ro (A.M.L.); cristinacos@yahoo.com (C.P.); 3 Endocrinology Department, University of Medicine and Pharmacy “Carol Davila”, 050474 Bucharest, Romania; 4 Endocrinology Department, National Institute of Endocrinology “C.I. Parhon”, 011863 Bucharest, Romania; 5 Carcinogenesis and Molecular Biology Department, Institute of Oncology “Prof. Dr. Alexandru Trestioreanu”, 022328 Bucharest, Romania; 6 Oncology Department, University of Medicine and Pharmacy “Carol Davila”, 050474 Bucharest, Romania; 7 Radiotherapy II Department, Institute of Oncology “Prof. Dr. Alexandru Trestioreanu”, 022328 Bucharest, Romania

**Keywords:** quantitative analysis, SPECT-CT, metastatic bone disease, qualitative imaging interpretation, follow-up SPECT-CT

## Abstract

(1) Background: Considering the importance that quantitative molecular imaging has gained and the need for objective and reproducible image interpretation, the aim of the present review is to emphasize the benefits of performing a quantitative interpretation of single photon emission computed tomography-computed tomography (SPECT-CT) studies compared to qualitative interpretation methods in bone lesion evaluations while suggesting new directions for research on this topic. (2) Methods: By conducting comprehensive literature research, we performed an analysis of published data regarding the use of quantitative and qualitative SPECT-CT in the evaluation of bone metastases. (3) Results: Several studies have evaluated the diagnostic accuracy of quantitative and qualitative SPECT-CT in differentiating between benign and metastatic bone lesions. We collected the sensitivity and specificity for both quantitative and qualitative SPECT-CT; their values ranged between 74–92% and 81–93% for quantitative bone SPECT-CT and between 60–100% and 41–100% for qualitative bone SPECT-CT. (4) Conclusions: Both qualitative and quantitative SPECT-CT present an increased potential for better differentiating between benign and metastatic bone lesions, with the latter offering additional objective information, thus increasing diagnostic accuracy and enabling the possibility of performing treatment response evaluation through accurate measurements.

## 1. Introduction

Nowadays, nuclear medicine represents an important tool in the diagnosis, monitoring, and even treatment of different diseases by providing a functional characterization of the interested tissues [1,2]. Until recently, the use of quantitative image interpretation was available only in positron emission tomography-computed tomography (PET-CT) studies due to their better spatial resolution and iterative reconstruction algorithms used for image processing which enabled the inclusion of attenuation correction maps based on transmission imaging techniques, such as computed tomography (CT) data [3]. The development of new technology has enabled the expansion of quantitative metrics, usually used in PET-CT image interpretation, to single photon emission computed tomography-computed tomography (SPECT-CT) [3,4,5]. The opportunity to use a wider range of radiopharmaceuticals for various applications and its lower cost and higher availability compared to PET-CT systems indicate that quantitative SPECT-CT can also provide valuable information for patient diagnosis and management [6,7]. Considering that bones represent the third most frequent site of metastases in cancer patients [8], one of the main applications of quantitative SPECT-CT is the evaluation of bone metastases, facilitating the differentiation between benign and malignant bone lesions [9]. The feasibility of performing quantitative interpretations of bone SPECT-CT examinations has been previously emphasized in several studies. Arvola et al. [10] demonstrated that the standardized uptake values (SUVs) registered by performing quantitative measurements on bone 99 m technetium-hydroxy diphosphonate ([^99m^Tc]-HDP) SPECT-CT studies strongly correlated to the ones registered on [^18^F]-sodium-fluoride (NaF) PET-CT. Beck et al. [11] also indicated that the usage of quantitative metrics in the image interpretation of [^99m^Tc]-HDP SPECT-CT data resulted in a significantly higher inter-observer agreement. Acknowledging this, it seems reasonable to assume that quantitative bone SPECT-CT interpretation could result in reproducible, objective image interpretation that can serve not only for better differentiation between benign and metastatic lesions but also for patient management and accurate treatment response evaluation [11,12,13,14]. The need to perform quantitative analysis has been demonstrated in the past by highlighting the significant variability and disagreement between observers when only a qualitative assessment of the SPECT-CT data was performed for follow-up purposes and by the relatively weak correlation between qualitative and quantitative image interpretation methods. Some of the advantages of quantitative SPECT-CT are its cost-effectiveness, wide availability, and established national reimbursement strategies, compared to PET-CT studies, with a similar accuracy to [^18^F]-NaF PET-CT as demonstrated by Arvola et al. [10]. Furthermore, quantitative methods express the degree of radiotracer accumulation, in the target lesions, in absolute values, thus offering a clear reference point for further comparisons between the scans, assuming that the acquisition and reconstruction of the data are performed using the same protocol [11,14].

The aim of this review is to assess the added value of quantitative bone SPECT-CT in cancer patients diagnosed with bone metastases and to identify new directions for extensive research on this subject.

## 2. Materials and Methods

A comprehensive electronic search to find relevant published literature was conducted in the PubMed and SCOPUS databases. The time interval considered for this analysis was 2003 to present and the databases were last searched on 10 July 2023. The Preferred Reporting Items for Systematic reviews and Meta-Analysis (PRISMA) methodology was used as a guideline to conduct this review. To perform a comprehensive search, the syntax was based on multiple forms and a combination of the terms “quantitative”, “bone metastases” “SPECT-CT”, “whole body SPECT-CT”, “osseous metastases”, “WB SPECT-CT”, and “treatment response” and “follow-up”.

### 2.1. Study Selection

Inclusion criteria comprised studies that: reported accuracy of bone metastases diagnostic using SPECT-CT; reported SUVs; used ^99m^Tc labeled bone-seeking agents, such as [^99m^Tc]-HDP/hydroxymethylene diphosphonate (HMDP) or [^99m^Tc]-methylene diphosphonate (MDP); conducted lesion and patient-based analyses; had a minimum of 10 patients to avoid selection bias; and were peer-reviewed and published in the English language.

**Figure 1:**
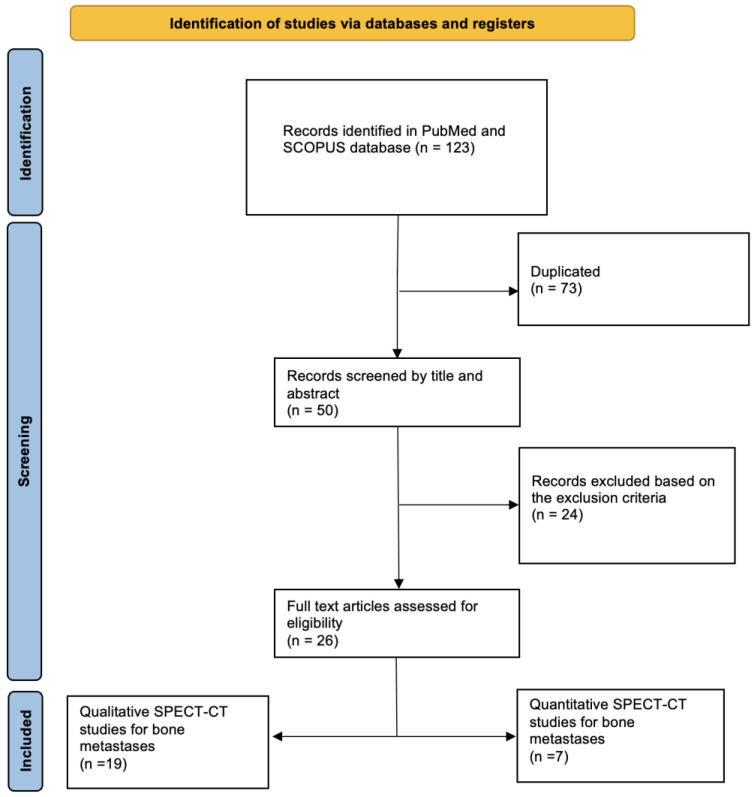
PRISMA representation of the process of the literature selection for this review.

Articles were excluded if they were performed for prognostic value, physics purposes, pathologies other than metastatic bone lesions, phantom or animal experiments, case reports, abstracts, or review articles with insufficient information on the study background or methodology, or duplicated (Figure 1).

The electronic search identified 123 papers on quantitative and qualitative diagnostic accuracy evaluation in bone metastases by SPECT-CT imaging, from which 73 were duplicates and 24 were excluded based on the specified criteria, thus resulting in a total number of 26 studies eligible for the present analysis.

### 2.2. Data Extraction

All data were evaluated independently by two reviewers, in terms of quality and bias risk, to ensure that every article complied with the inclusion criteria. The following information regarding the included studies was obtained: author names, year of publication, study design, sample size, number of lesions, SUVs, description of patient samples, scanner type, radiotracer, administered dose, injection scan time, attenuation correction, and imaging reconstruction methods.

The area under the curve (AUC), sensitivity, and specificity for both qualitative and quantitative studies were registered when available. In addition, cut-off values were also registered for quantitative bone SPECT-CT.

## 3. Results

### 3.1. Study Characteristics and Diagnostic Accuracy of Qualitative SPECT-CT

#### 3.1.1. Study Characteristics

Table 1 summarizes the main characteristics of the studies focused on qualitative SPECT-CT in metastatic bone lesion evaluations. All authors included in this review used different scales as a semiquantitative method for lesion characterization, the most common being the 5-point scale from 0 to 4, where 0 represented normal lesions and 4 malignant lesions.

The concept of diagnostic accuracy analysis in hybrid SPECT-CT images has been around for almost two decades. One of the first studies was conducted by Utsunomyia et al. [15] on 45 cancer patients and aimed to assess the additional diagnostic value of fused SPECT and CT images in metastatic bone lesion evaluations. The conclusion was that the hybrid SPECT-CT provides information that can help differentiate between benign and malignant bone lesions.

Mahaletchumy et al. [32] focused on the potential benefit of bone SPECT-CT in detecting skeletal abnormalities in patients with breast cancer. The authors analyzed the data of 85 patients and concluded that SPECT-CT significantly improved both sensitivity and specificity in diagnostic bone metastases while reducing the proportion of equivocal findings.

Zhao et al. [16] compared the diagnostic efficacy of SPECT-CT fusion imaging with that of SPECT alone and with SPECT + CT. The study included 125 cancer patients, mainly diagnosed with lung and breast cancer. The conclusions of their study were that SPECT-CT showed good performance in assessing equivocal lesions visible on SPECT or CT, specifically located in the spine and ribs, improved the accuracy in image interpretation, and shorted the diagnostic process.

In 2014 Sharma et al. [18], in a study with 32 cancer patients, evaluated the diagnostic accuracy of SPECT-CT in the characterization of isolated skull lesions in comparison with bone scintigraphy and Palmedo et al. [17] analyzed a more significant number of patients, 325 (211 with breast cancer, 97 with prostate cancer), assessing the added value of the SPECT-CT of the trunk, when used in conjunction with conventional nuclear medicine techniques, and its effects on patient management. Both studies strengthened the findings of Zhao et al. [16] by showing the added value of SPECT-CT in equivocal lesions classification.

In 2015, Zhang et al. [19], in a study with 65 hepatocellular carcinoma (HCC) patients confirmed through biopsy, evaluated the incremental value of SPECT-CT for equivocal bone lesion assessments in hepatocellular carcinoma; their results recommended the use of planar bone imaging combined with SPECT-CT when encountering suspicious bone lesions in these type of patients.

Three studies, conducted by Rager et al. [24], on 212 patients presenting various cancers, Mavriopoulu et al. [25], on 257 patients (256 breast cancer and 1 prostate cancer patient), and Fleury et al. [27], on 328 patients (164 breast cancer and 164 prostate cancer patients), evaluated the diagnostic accuracy of bone SPECT-CT performed in a systematic manner. The results indicated that systematic bone SPECT-CT from the cervical region to the proximal femur should be the modality of choice in investigating bone lesions, observing a significantly improved diagnostic accuracy with the small cost of a longer acquisition time and slightly higher radiation dose administered to the patient.

In 2020, Zhang et al. [31] tried to establish a grading diagnostic criteria for bone metastases evaluation, based on the data collected from 100 patients presenting various cancers. The authors could not identify the appropriate diagnostic criteria to ensure both high interviewer agreement and specificity in lesion characterization.

One of the most interesting studies regarding bone metastases evaluation was performed by Zhang et al. [33] in 2022, with a study population of 74 prostate cancer patients. The authors compared the diagnostic accuracy of [^99m^Tc]-MDP SPECT-CT and [^99m^Tc]-PSMA and concluded that [^99m^Tc]-PSMA SPECT-CT had a higher sensitivity and specificity than [^99m^Tc]-MDP SPECT-CT in terms of bone metastasis detection in prostate cancer patients.

Several authors performed prospective studies comparing bone SPECT-CT with PET-CT diagnosis capabilities: Löfgren et al. [21] conducted a study of 97 cancer patients (62 men with prostate cancer, 54 women with breast cancer, and 1 woman with renal cancer), 53 high-risk cancer patients (26 breast and 27 prostate) were studied by Jambor et al. [20], 39 prostate cancer patients by Fonager et al. [22], and 213 prostate cancer patients in the study conducted by Dyrberg et al. [28]. The conclusions were as follows: Fonager et al. [22] demonstrated that SPECT-CT had comparable diagnostic capacity to [^18^F]-NaF PET-CT in high-risk prostate and breast cancer patients; Jambor et al. [20] concluded that SPECT-CT is indeed a good option for bone lesion evaluation, especially because of its economic implications compared to PET-CT and shorter acquisition times compared to DWI MRI; Löfgren et al. [21] observed that both [^18^F]-NaF PET-CT and SPECT-CT produce a significantly lower number of equivocal readings; and Dyrberg et al. [28] concluded that the diagnostic performances of [^18^F]-NaF-PET/CT, [^18^F]-Choline-PET-CT, WB-SPECT-CT and whole-body MRI were favorable for the detection of bone metastases in patients with prostate cancer.

Further comparison studies between bone SPECT-CT and PET-CT were performed only on prostate cancer patients. Janssen et al. [23], in 2017, conducted a study that included 54 prostate cancer patients with a mean prostate-specific antigen (PSA) level of 38.4 ± 77.9 ng/mL and mean age of 69.6 ± 6.5 years, while Simsek et al. [30], in 2020, enrolled 138 prostate cancer patients with a median PSA level of 18.3 ng/mL and mean population age of 66 years, with the same purpose, to compare the diagnostic abilities of bone SPECT-CT and ^68^Gallium-prostate specific membrane antigen ([^68^Ga]-PSMA) PET-CT in metastatic bone lesion identification and staging. The two groups concluded that performing [^68^Ga]-PSMA PET-CT significantly increased the diagnostic accuracy compared to bone SPECT-CT. Regarding ^18^Fluorine ([^18^F])-Choline, a study that included 115 prostate cancer patients, performed in 2020 by Leiris et al. [29], investigated whether bone SPECT-CT provides additional diagnostic information over [^18^F]-Choline PET-CT for detecting bone metastases in the setting of prostate cancer biochemical recurrence. The authors concluded that bone SPECT-CT does not provide additional diagnostic information over [^18^F]-Choline PET-CT.

#### 3.1.2. Diagnostic Accuracy

By performing receiver operating characteristic (ROC) statistical analysis, the authors obtained data regarding the diagnostic performances of bone SPECT-CT in bone metastases evaluation. The indexes used to express the diagnostic abilities of bone SPECT-CT are the area under the curve (AUC), sensitivity, and specificity (Table 2). The studies have shown a great improvement, compared to conventional molecular imaging techniques, in diagnostic performance in evaluating bone lesions when fused images were used. This can be clearly seen in the present review and even in the first study included, conducted by Utsunomyia et al. [15], where the authors obtained an AUC index of 0.96; unfortunately, however, they did not report the sensitivity and specificity. An interesting aspect is that a high AUC was obtained even if the registration of the images was done manually and the SPECT data and CT data were acquired on separate scanners, meaning that, in some cases, the position of the patient might have significantly changed between the scans. Nevertheless, considering that the method for image interpretation was a qualitative evaluation, this aspect was not so relevant to the outcome of the evaluation.

Further studies focused only on bone SPECT-CT diagnostic accuracy. The results were in agreement with the ones determined by Utsunomyia et al. [15] in 2005. The AUC sensitivity and specificity of these studies were as follows: Zhao et al. [16] 0.95, 98%, and 93% in a very heterogeneous group of patients; Zhang et al. [19] 0.99, 100%, and 97%, in HCC patients, and Mahaletchumy et al. [32], who included only breast cancer patients, which tend to present lytic bone metastases rather than sclerotic lesions, which is one of the possible explanations for the lower values of sensitivity in their group, 78%, but with a comparable specificity of 94%; the authors did not report the AUC value.

Even in situations when bone lesions localized in specific regions were evaluated, the studies conducted by Sharma et al. [18] and Palmedo et al. [17], or aimed to determine the added value in performing systematic bone SPECT-CT scans in metastatic bone patients, in the studies by Zhang et al. [19] 2015, Mavriopoulu et al. [25], and Fleury et al. [27], the AUC ranged from 0.89 to 0.99, with a sensitivity between 96 and 100% and specificity between 94 and 98%. All these demonstrate a wide range of applications that SPECT-CT could possess when evaluating bone lesions in different settings and analyzing data from patients diagnosed with various types of cancer.

Only one study, conducted by Rager et al. [24] in 2018, compared the diagnostic accuracy of bone SPECT-CT with [^18^F]-FDG PET-CT. The study included 25 histologically proven female breast cancer patients. Their results showed a sensitivity and specificity for bone SPECT-CT of 95% and 100%, compared to 83% and 100% for [^18^F]-FDG. The authors concluded that SPECT/CT may be a useful adjunct to [^18^F]-FDG PET/CT for the staging of breast cancer patients.

Zhang et al. [31] in 2020 and again in 2022 obtained a sensitivity of 100% and a specificity of 41%, respectively, an AUC, sensitivity, and specificity of 0.84, 72%, and 81% for [^99m^Tc]-MDP in bone lesion evaluations. The low specificity registered in their study in 2020 can be explained by the highly heterogeneous patient cohort included and the significant percentage of lytic lesions, 77.8%.

One of the most notable aspects of the diagnostic accuracy of bone SPECT-CT is the good results compared to PET-CT studies using different radiotracers, in which the AUC, sensitivity, and specificity values ranged between 0.92 and 0.95, 60 and 100%, and 92 and 100%, compared to 0.96, 92 and 100%, and a specificity of 90–97% when [^18^F]-NaF was used for comparison (Jambor et al. [20], Fonager et al. [22], Löfgren et al. [21], and Dyrberg et al. [28]); data regarding the PET-CT AUC were only reported by Jambor et al. [20].

When comparing bone SPECT-CT diagnostic accuracy to [^68^Ga-PSMA] or [^18^F]-Choline, used in prostate cancer patient management (Janssen et al. [23], Dyrberg et al. [28], Leiris et al. [29] and Simsek et al. [30]), the results indicated that the AUC, sensitivity, and specificity of bone SPECT-CT ranged between 0.82 and 0.89, 82 and 100% and 82 and 94%, compared to 0.82 and 1.00, 93 and 100% and 95 and 100%. 

### 3.2. Study Characteristics and Diagnostic Accuracy of Quantitative SPECT-CT

#### 3.2.1. Study Characteristics

Table 3 summarizes the main characteristics of the studies focused on quantitative SPECT-CT in metastatic bone lesion evaluations.

The first quantitative study, published in 2017, was retrospectively performed by Kuji et al. [34] and aimed to determine the diagnostic accuracy of quantitative SPECT-CT in differentiating between benign and metastatic bone lesions. The study included 170 prostate cancer patients presenting 126 metastatic bone lesions, with a mean injected activity and injection-scan time of 850 ± 168 MBq and 204 ± 27 min. The SPECT scanning parameters included 60 views with 10 s/view.

In 2019, another retrospective study with the same target was conducted by Tabotta et al. [35] on a cohort of 39 prostate cancer patients presenting a total of 265 metastatic lesions. The SPECT/CT protocol was performed with 120 views and 12 s/view. We observed that although they registered comparable values for injected activity and injection-scan time, a significantly higher number of views for the SPECT data acquisition were considered, resulting in a higher image quality of the target lesions.

Rohani et al. [36], in a paper published in 2020, evaluated 34 prostate cancer patients presenting 122 metastatic lesions, a significantly lower number of lesions evaluated in comparison to Tabotta et al. [35] but with a relatively similar number of patients. However, the data referring to the scanning parameters and image reconstruction algorithms were scarce.

In another retrospective study published in 2021, Zhang et al. [31] evaluated, for the first time using quantitative SPECT-CT, a heterogenous sample of 51 patients presenting just 48 bone metastatic lesions, indicating that the results from the study may not be sufficient to achieve statistical significance.

In 2022, Gherghe et al. conducted the first prospective study for evaluating the diagnostic accuracy of quantitative SPECT-CT in discriminating between benign and metastatic bone lesions. This study focused on breast cancer and included a total of 70 patients, encompassing 236 metastases. The SPECT-CT scans were conducted using a 60-view acquisition protocol with a duration of 20 s per view. Additionally, iterative image reconstruction methods were employed, utilizing eight subsets and 10 iterations. Therefore, the collected data can be regarded as accurate, both in terms of the acquisition protocol and reconstruction methods applied.

In the most recent retrospective study, published in 2023 by Lin et al. [39], the authors evaluated the diagnostic accuracy of quantitative SPECT/CT in differentiating between benign and metastatic bone lesions in patients diagnosed with lung adenocarcinoma. The study included 115 male and female patients presenting 252 bone metastases. No data were available for the injection-scan time parameter which represents an important issue because of their influence on the SUVs.

When optimizing quantitative SPECT-CT, it is essential to consider numerous factors related to images, the controllable (acquisition time, collimator, matrix size, SPECT orbit radius, number of iterations and subsets, and post-reconstruction filtering) and uncontrollable (dead-time, lesion/organ size and shape, organ-to-background contrast, organ location, and patient movement). According to the EANM practice guideline, bone disease represents the leading clinical application for quantitative SPECT/CT with the potential to improve reader diagnostic certainty and treatment response evaluation [40,41].

All the studies performed attenuation correction of the raw data and employed different protocols for image reconstruction algorithms, rotation degree values, and time per view for the “step and shoot” technique. This highlights the importance of establishing standardized image acquisition protocols to ensure that comparisons are relevant when conducting meta-analyses of existing data.

It has been demonstrated on numerous occasions that the total number of iterations used for imaging reconstruction has a significant impact on the SUV measurement. From the present data, we can see that similar numbers of iterations were used in the studies conducted by Kuji et al. [34] and Zhang et al. [31], while the highest number of iterations is seen in the study conducted by Gherghe et al. [37]. The lowest number of iterations is seen in the most recent study conducted by Lin et al. [39], while Tabotta et al. [35] and Rohani et al. [36] did not provide these data for comparison. The present aspect, in addition to the injection-to-scan time, has a great impact on the final SUVs. It has been previously demonstrated that a higher number of iterations results in a higher accuracy of the measurements, with the downfall of prolonged computation time [40,41].

An important point to highlight is the heterogeneity in the scanning protocols and image reconstruction algorithms that existed prior to the publication of the EANM guideline. This variability has posed challenges when attempting to conduct a meta-analysis.

#### 3.2.2. Diagnostic Accuracy

Regarding the results obtained from the studies included in the present review, we can observe that the SUVmax values ranged from 23.8 g/mL, registered by Lin et al. [39]., to 40.9 g/mL, resulting from the study by Kuji et al. [34]. Focusing on the cut-off values used to discriminate the benign from metastatic lesions, we can observe that a significantly lower value, of just 11.1 g/mL, resulted in metastases from pulmonary adenocarcinoma in the study by Lin et al. [39], compared to the cut-off values determined by the other authors, which ranged between 16.6 g/mL, obtained by Gherghe et al. [37], and 20.0 g/mL, measured by Rohani et al. [36]. One explanation for this may be the lower SUVmax of bone metastases in patients with lung adenocarcinoma than in patients with prostate and breast cancer. Unfortunately, the paper by Kuji et al. [34] does not mention the calculation of cut-off values.

The SUVmax of benign bone lesions was available for comparison in each study, presenting lower values than the SUVmax of the metastatic bone lesions (Table 2 and Table 3). All authors demonstrated that there was indeed a statistically significant difference between benign and metastatic bone lesions in terms of radiotracer uptake, which translates into higher SUVmax values for the latter group.

The ROC statistical analysis registered high values regarding the diagnostic accuracy of the quantitative SPECT-CT in discriminating between benign and metastatic lesions, with the lowest value of 0.687 registered by Zhang et al. [31], while the highest AUC value of 0.974 was registered by Gherghe et al. [37] (Table 2).

A noteworthy observation is that the groups with the highest number of lesions, Tabotta et al. [35], Gherghe et al. [37], Ikeda et al. [38], and Lin et al. [39], achieved the most elevated AUC, with consistent cut-off values. The ability to distinguish between benign and metastatic bone lesions using quantitative SPECT-CT seems to be more accurate due to the large amount of statistical data available for comparison.

Sensitivity and specificity were available for comparison in only five studies, presenting variable values for both features, with sensitivity ranging from 74%, by Rohani et al. [36], to 92%, by Gherghe et al. [37], and specificity between 81%, by Lin et al. [39], and 93%, also by Gherghe et al. [37] and Ikeda et al. [38] (Table 4).

Analyzing the pooled mean value of SUVmax resulting from the studies included in this review, regardless of the type of cancer, the majority of bone lesions presenting an SUVmax value greater than 32.2 g/mL are most likely to be categorized as malignant, while lesions with an SUVmax lower than 13.2 g/mL are, in most cases, considered benign (Table 5).

### 3.3. Study Parameters and Benefits for Bone Metastasis Treatment Response Evaluation by Quantitative SPECT-CT

Regarding the potential use of quantitative SPECT-CT for follow-up purposes in evaluating metastatic bone lesions, we can observe that, between 2016 and 2023, no new data were published in the literature. The only studies available for comparison are a retrospective study conducted by Beck et al. [11], which included 19 patients, from which 16 were female patients presenting breast cancer and 3 were male patients presenting prostate cancer, and a prospective study conducted by Gherghe et al. [14] which included 75 female breast cancer patients.

The number of metastatic lesions evaluated by Beck et al. [11] was 52, scanned at an interval of 3–24 months, while Gherghe et al. [14] evaluated 249 lesions at an interval of 6–8 months after the initial scan. There were no significant changes in protocol parameters such as injected activity and injection-scan time (Table 6).

By analyzing the data present in Table 7 from the study conducted by Beck et al. [11], we can determine that the qualitative evaluation from both planar scans and SPECT/CT images registered only a fair to moderate inter-observer agreement with a k of 0.46 and 0.35, respectively. In comparison, when a quantitative SPECT-CT evaluation was performed, the inter-observer agreement was almost perfect, with a k of 0.94. Furthermore, by looking at the results obtained by Gherghe et al. [14], there is a moderate correlation between qualitative whole-body planar scintigraphy (WBS) and quantitative SPECT-CT in terms of treatment response evaluation, supported by a correlation coefficient of 0.608, and a strong positive correlation between qualitative and quantitative SPECT-CT, showcasing a correlation coefficient of 0.711. However, in a significant number of cases, inconsistent results were obtained between qualitative and quantitative interpretation methods (Table 5).

## 4. Discussion

Commonly, SPECT-CT has been used as a qualitative method for the evaluation of various diseases, but the progressive development of image reconstruction algorithms, attenuation correction maps, and scatter correction techniques have made possible the quantitative analysis of SPECT-CT images [42].

After the emergence of quantitative SPECT-CT, some significant changes have been made regarding the content of the methods section reported in quantitative compared to qualitative SPECT-CT studies. The first aspect is that the injected activity, height, weight, gender, injection-scan time, and exact dose of the injected radiotracer need to be included, along with the number of iterations and subsets used for image reconstruction. Another aspect is that the scanner needs to be properly calibrated [41] to ensure accurate measurements of the activity in the region of interest.

In terms of image interpretation, compared to qualitative analysis, when the quantitative method was used, the authors were able to choose a standard of reference to identify the pathological accumulation of the radiotracer, most frequently either a normal thoracic or lumbar vertebra [31,34,38], while on numerous occasions, when qualitative interpretation was applied, choosing such references was difficult.

Although qualitative SPECT-CT has great diagnostic value in evaluating bone metastases, many benign lesions can manifest as bone-destructive entities, concentrating high amounts of the radiotracer and, thus, translating into false-positive results. Conventional SPECT-CT lacks objectivity and reproducibility, making it difficult to directly compare the results obtained in different studies [31]. The added value of quantitative SPECT-CT in this situation is that, for quantitative purposes, some clear steps need to be performed to obtain accurate measurements, thus opening the possibility of implementing a general acquisition protocol across all clinics and a standard procedure in SPECT-CT quantification. Another situation when quantitative SPECT-CT shows its value is when atypical bone lesions on CT findings are identified or when radiotracer foci are seen with SPECT with no obvious morphological correspondence on the CT images [39].

When performing qualitative analysis, the imaging diagnosis is largely dependent on the reviewers’ experience; therefore, by incorporating quantitative measurements, more diagnostic information is available, thus increasing the diagnostic accuracy.

Based on the currently available data, a general conclusion is that performing a quantitative analysis can help differentiate active bone metastases from benign lesions in patients with prostate, breast, and other types of cancer. Additionally, it is important to emphasize the significance of objective data in patient evaluation and that quantitative SPECT-CT has the potential to become a dependable osteoblastic biomarker [31,34,39].

A review conducted by Ross et al. [9] in 2019 stated that the addition of SUV metrics in SPECT-CT could help in delivering information on radiotracer uptake with greater objectivity while also having a potential impact on patient management.

The first instance has been gradually demonstrated throughout various studies, such as Beck et al. [11] who showed that quantitative SPECT-CT presented almost perfect inter-observer agreement in image interpretation while qualitative interpretation methods yielded only moderate inter-observer agreement. Notably, the study by Gherghe et al. [19] recorded the highest AUC value of 0.974, with a sensitivity of 92% and specificity of 93% at a cut-off value of 16.6 g/mL. This highlights the high accuracy achieved by quantitative methods in this context. These findings further support and bring us closer to the objective of quantitative SPECT-CT to offer information that can help differentiate between benign and malignant lesions [9,43].

In a study published in 2019 by Arvola et al. [10], the authors demonstrated that, similar to PET-CT imaging, the SUVs resulting from the SPECT-CT quantitative analysis can represent a reliable tool for the bone metabolism assessment of osseous metastases in breast and prostate cancer patients by performing a correlation between the SUVs resulting from SPECT-CT using [^99m^Tc]-HDP and [^18^F]-NaF PET-CT.

Various cut-off values, AUC, sensitivity, and specificity for bone SPECT-CT have been registered, facilitating the potential for better utilization of the quantitative SPECT-CT in clinical practice. This statement is supported by the fact that the cut-off values are relatively homogenous, with the lowest value being 11.1 g/mL, reported by Lin et al., and the highest being 20 g/mL, reported by Rohani et al. [36] This difference can be explained by the fact that in lung adenocarcinoma, bone metastases may be either osteoblastic, osteolytic or mixed lesions, expressing a variable radiotracer uptake because of the higher affinity of diphosphonates for sites with accentuated bone tissue synthesis compared to the ones where the process is mostly lytic, in which cases, the bone formation processes are reduced, presenting lower SUVs [44,45]. Nevertheless, similar to PET-CT [46], establishing the appropriate cut-off values for SUVs in target lesions is a complex task. Halim et al. [47] elucidated that SUVs can be influenced by diverse physical and biological factors, including partial volume and spill-over effects, attenuation correction, reconstruction methods, and parameters specific to the scanner type, count noise bias effect, time elapsed between radiotracer injection and imaging, body size, and more. Standardizing SPECT-CT protocols can help mitigate SUV variability and enhance the reliability of SUV thresholds across different institutions [41]. Another factor to be considered when calculating SUVs is that different values for the same lesion can arise when different radiotracers are used, as demonstrated by Arvola et al. [10]. This discrepancy can be attributed to the distinct pharmacokinetics and dynamics of these radiotracers. Consequently, based on the findings from their study, the authors concluded that it is not advisable to directly translate PET SUVs into SPECT SUVs, implying that SPECT studies should not be used as a substitute for PET in follow-up examinations for the same patient [10].

Conventional bone scintigraphy utilizing bone-seeking agents exhibits acceptable sensitivity with only moderate specificity. The inclusion of SPECT substantially improves the accuracy of detecting metastatic bone lesions, and this accuracy is further enhanced by incorporating [^18^F]-NaF PET-CT imaging. A study conducted by Sapir et al. [48] demonstrated a good sensitivity of 92% and specificity of 82% for metastatic lesion detection in SPECT compared to [^18^F]-NaF PET (sensitivity of 100% and 62% specificity).

In a phase III trial conducted in Canada (MITNEC-A1), Bénard et al. [49] discovered that [^18^F]-NaF PET-CT exhibited a greater accuracy (84.3%) when detecting skeletal metastases compared to [^99m^Tc]-MDP SPECT (77.4%) in intraindividual comparisons. This study specifically focused on patients with high-risk prostate or breast cancers.

In recent years, the advancement of PET-CT radiotracers has provided reliable means for evaluating metastatic bone lesions with high levels of sensitivity and specificity. When assessing the diagnostic performance of FDG PET-CT in detecting metastatic bone lesions, Yang et al. conducted a meta-analysis and reported a pooled sensitivity ranging from 90% to 96% and a specificity of 96% to 98% [50]. In comparison, Alqahtani et al. investigated the diagnostic performance of bone SPECT-CT and found a sensitivity ranging from 89% to 94% and a specificity of 94% to 97% [8]. These results indicate that both methods exhibit comparable diagnostic performance for evaluating bone metastases.

Furthermore, studies have shown that [^18^F]-NaF has a sensitivity ranging from 95% to 98% and a specificity of 81% to 87% [51], which are similar to the findings of Alqahtani et al. This highlights that SPECT-CT utilizing [^99mTc^]-HDP remains a viable option for evaluating metastatic bone lesions.

When considering the diagnostic accuracy of quantitative analysis, an intriguing observation arises regarding the relatively lower value reported by Zhang et al. [31] in comparison to other authors. One possible explanation is that their study included a highly heterogeneous group of patients, whereas the other authors primarily focused on evaluating the value of quantitative SPECT-CT in metastases from a single type of cancer [52,53,54,55,56].

Regarding the SUVs that can be obtained by performing quantitative SPECT-CT, the only parameters that were calculated using the threshold function of the segmentation tools were the SUVmax and SUVmean [48], but, with the development of new reconstruction algorithms and segmentation software, the SUVpeak has become available for measurements in a fully automatic manner, thus making quantitative SPECT-CT even closer to the quantitative PET-CT imaging standards. This indicates a significant advancement, as the SUVpeak combines the accuracy of the SUVmax with the low variability of the SUVmean, thereby enabling precise measurements to be conducted consistently across various research centers [13,14,57].

Furthermore, Gherghe et al. [14] emphasized the need for quantitative image interpretation methods by demonstrating that while there is a strong correlation between qualitative and quantitative SPECT-CT in terms of treatment response evaluation, approximately 30% of the cases yielded inconsistent results. This underscores the additional value provided by quantitative SPECT-CT in patient management.

Considering the results of the ROC statistical analysis, we can observe that the AUC, sensitivity, and specificity of the qualitative bone SPECT-CT range between 0.82 and 1.00, 60 and 100%, and 41 and 100%, while for quantitative bone SPECT-CT, the values range between 0.68 and 0.97, 74 and 92% and 81 and 92%. Although slightly lower values resulted from the quantitative studies, it is important to carefully analyze the results obtained from the qualitative ones. The wide range of values, regarding the diagnostic parameters, is one of the aspects that shows how much the results depend on the reviewer’s experience when the qualitative method is used. We also consider that one of the possible explanations for obtaining the 100% “perfect” results can be found in the subjectiveness of the characterization criteria used by each group for lesion evaluation. As expected, the quantitative interpretation method had more homogeneous results because of the objectiveness of the data; thus, it seems reasonable to assume that it could represent a more reliable and reproducible method compared to qualitative image interpretation.

Although the qualitative and quantitative analyses have comparable diagnostic accuracies in evaluating bone lesions, because the quantitative assessment offers additional diagnostic information that can help correctly characterize lesions, it seems reasonable to assume that it represents a powerful tool in bone lesion evaluation that can serve as a surrogate whenever PET-CT is not available, and with lower costs and wider availability. One of the most convincing aspects that shows the increasing value of quantitative SPECT-CT is that the European Association of Nuclear Medicine (EANM) has recently published a dedicated guideline on quantitative SPECT-CT [43] which encompasses data on the potential applications of quantitative SPECT-CT, such as: (1) delivering a reliable diagnosis, (2) accurate therapy response monitoring, (3) prognosis and guiding patient management decisions, (4) improving the reproducibility of interpretations, (5) allowing the comparison of data between centers, (6) and facilitating (semi)automatic analysis [41]. The authors of the guideline point out that one of the main applications for quantitative SPECT-CT is represented by bone lesion evaluations [41].

## 5. Limitations

As anticipated, the current analysis has limitations that include the scarce and heterogeneous nature of the data collected from each study. Additionally, a significant challenge in conducting a meaningful meta-analysis of quantitative SPECT-CT studies on metastatic bone lesions is the absence of international guidelines for image acquisition and reconstruction protocols. This results in different configurations for each gamma camera used for scanning, which can significantly affect the SUVs. Another limitation is represented by the unavailability of sufficient statistical data to perform a meta-analysis regarding the diagnostic capacities of the two methods of image interpretation.

## 6. Conclusions

To summarize, both qualitative and quantitative SPECT-CT have shown their potential in distinguishing between metastatic and benign bone lesions. The increasing value of quantitative SPECT-CT in clinical practice is represented by the objective information on radiotracer uptake in target lesions which can play an important role in treatment response evaluation, especially, and when performing personalized medicine. Furthermore, it can offer a robust, precise, and dependable tool in dosimetry and bone disease characterization. Therefore, it seems reasonable to assume that there is an urgent need for future research studies conducted in multiple centers on metastatic bone patients using standardized acquisition protocols and analyzing the same parameters. These data could generate clear and reliable conclusions on the actual impact that SPECT-CT can have in daily clinical practice.

## Figures and Tables

**Table 1:** General data from the SPECT-CT qualitative studies.

Authors	Year of Publication	Study Design	Cancer Type	Patients (n)	Injected Activity (MBq)	Injectio-Scan Time (min)	Image Correction Applied	Image Reconstruction Method (Subsets and Iterations)	Scanner Type	Radiotracer
Utsunomiya et al. [15]	2005	R	N/A	45	555	120–180	No	No	Juxtaposed devices	^99m^Tc -MDP/HDP
Zhao et al. [16]	2010	N/A	BC and LC	125	740	180	Yes	OSEM (4/8)	Philips Precedence	^99m^Tc-MDP
Palmedo et al. [17]	2014	P	BC and PC	353	643–712	120–240	N/A	N/A	GE Hawkeye 4 and Siemens Symbia T2	^99m^Tc-MDP
Sharma et al. [18]	2014	R	VC	32	666–925	180	Yes	Flash 3D (8/8)	Siemens Symbia T6	^99m^Tc-MDP
Zhang et al. [19]	2015	R	HCC	65	925–1110	180–360	N/A	N/A	Prism-IRIX Marconi	^99m^Tc-MDP
Jambor et al. [20]	2016	P	BC and PC	53	670	180	Yes	Flash 3D (5/10)	Siemens	^99m^Tc-HDP
Löfgren et al. [21]	2017	P	BC, PC, and RC	117	523–655	180	Yes	Flash 3D(5/10) and Astonish (4/16)	Siemens Symbia T16 and Precedence Philips	^99m^Tc-HDP
Fonager et al. [22]	2017	P	PC	37	750–1000	120–180	N/A	N/A	Siemens Symbia T2 and T16	^99m^Tc-MDP
Janssen et al. [23]	2017	R	PC	54	654	150	N/A	N/A	Siemens Symbia T6	^99m^Tc-DPD
Rager et al. [24]	2017	N/A	VC	212	400–1500	180	Yes	Flash 3D (8/8)	Siemens Symbia T6	^99m^Tc-HMDP
Mavriopoulu et al. [25]	2018	P	BC	257	630–700	180	Yes	OSEM(2/10)	GE Hawkeye and Hawkeye-4	^99m^Tc-HDP
Rager et al. [26]	2018	R	BC	25	N/A	180	Yes	Flash 3D (8/8)	Siemens Symbia T6	^99m^Tc-HDP
Fleury et al. [27]	2018	R	BC and PC	328	N/A	180	Yes	OSEM (4/8)	GE Discovery NMCT 670 and Simens Symbia T2	^99m^Tc-MDP
Dyrberg et al. [28]	2018	P	PC	213	N/A	N/A	N/A	N/A	Philips Precedence	N/A
Leiris et al. [29]	2020	R	PC	115	550	180	Yes	OSEM (5/8)	Siemens Symbia T2	^99m^Tc-MDP
Simsek et al. [30]	2020	R	PC	102	740	180	Yes	N/A	GE Discovery NM 630 and NM/CT 670	^99m^Tc-MDP
Zhang et al. [31]	2020	R	VC	120	1110	180–360	N/A	N/A	Prism-IRIX Marconi, Siemens Intevo, and Philips Precedence	^99m^Tc-MDP
Mahaletchumy et al. [32].	2022	P	BC	85	740	180	Yes	OSEM (2/10)	GE Hawkeye	^99m^Tc-MDP
Zhang et al. [33]	2022	P	PC	74	740	180–300	N/A	N/A	GE Discovery NM/CT 670Pro	^99m^Tc-MDP

N/A—not available; PC—prostate cancer; VC—various cancer; BC—breast cancer; LC—lung cancer; HCC—hepatocellular cancer; R—retrospective; and P—prospective; OSEM—ordered subset expected maximization; ^99m^Tc—99metastable technetium; HDP—hydroxy diphosphonate; MDP—methylene diphosphonate; DPD—3,3-diphosphono-1,2-propanodicarboxylicacid; HMDP—hydroxymethylene diphosphonate.

**Table 2:** Diagnostic accuracy of qualitative SPECT-CT in bone lesions in different settings.

Author	SPECT-CT			PET-CT			
	Area under the Curve (AUC)	Sensitivity (%)	Specificity (%)	Area under the Curve (AUC)	Sensitivity (%)	Specificity (%)	Radiotracer
Utsunomyia et al. [15]	0.96	N/A	N/A	N/A	N/A	N/A	N/A
Zhao et al. [16]	0.95	98	93	N/A	N/A	N/A	N/A
Sharma et al. [18]	1.00	95	100	N/A	N/A	N/A	N/A
Palmedo et al. [17]	0.91	97	94	N/A	N/A	N/A	N/A
Zhang et al. [19]	0.99	100	97	N/A	N/A	N/A	N/A
Jambor et al. * [20]	0.92	89	94	0.96	95	97	[^18^F]-NaF
Fonager et al. * [22]	N/A	89	100	N/A	89	90	[^18^F]-NaF
Rager et al. [24]	0.95	86	100	N/A	N/A	N/A	N/A
Löfgren et al. * [21]	N/A	60	92	N/A	80	97	[^18^F]-NaF
Janssen et al. * [23]	0.83	82	84	1.00	100	100	[^68^Ga]-PSMA
Dyrberg et al. * [28]	N/A	100	94	N/A	95/97	93/99	[^18^F]-NaF/[^18^F]-Choline
Fleury et al. [27]	N/A	97	98	N/A	N/A	N/A	N/A
Mavriopoulu et al. [25]	0.89	96	87	N/A	N/A	N/A	N/A
Rager et al. * [26]	N/A	95	100	N/A	83	100	[^18^F]-FDG
Leiris et al. * [29]	0.82	86	98	0.82	93	100	[^18^F]-Choline
Simsek et al. * [30]	0.89	95	82	0.96	97	95	[^68^Ga]-PSMA
Zhang et al. [31]	N/A	100	41	N/A	N/A	N/A	N/A
Mahaletchumy et al. [32]	N/A	78	94	N/A	N/A	N/A	N/A
Zhang et al. [33]	0.84	72	81	N/A	N/A	N/A	N/A

*—PET-CT comparison studies.

**Table 3:** General data from the SPECT-CT quantitative studies.

Authors	Year of Publication	Study Design	Cancer Type	Patients/Metastatic Lesions (n)	Injected Activity (MBq)	Injectio-Scan Time (min)	Coverage	SPECT Scanning Parameters (Rotation° and s/Step)	Image Correction Applied	Image Reconstruction Method (Subsets and Iterations)	Scanner Type	Radiotracer
Kuji et al. [34]	2017	R	PC	170/126	850 ± 168	204 ± 27	cervical-to-thoracic and/or lumbar-to-pelvic	6/10	Yes	CGZAS (1/48)	Siemens Symbia Intevo	^99m^Tc-MDP
Tabotta et al. [35]	2019	R	PC	39/265	777 ± 113	215 ± 54	regions with high uptake on planar scintigraphy	3/12	Yes	N/A	Siemens Symbia Intevo	^99m^Tc-DPD
Rohani et al. [36]	2020	R	PC	34/122	N/A	180	N/A	6/15	Yes	N/A	GE Discovery SPECT/CT NM 670	^99m^Tc-MDP
Zhang et al. [31]	2021	R	VC	51/48	899 ± 44	302 ± 51	selected to include the indeterminate foci from planar whole-body scintigraphy	N/A	Yes	OSGC+ (2/24)	Siemens Symbia Intevo	^99m^Tc-MDP
Gherghe et al. [37]	2022	P	BC	70/236	674 ± 57	176 ± 34	cervical-to-pelvic/lumbar-to-pelvic	6/20	Yes	OSEM (8/10)	GE Discovery D670	^99m^Tc-HDP
Ikeda et al. [38]	2022	R	BC and PC	147/64	N/A	180–240	N/A	3/15	Yes	OSEM (10/10)	Ge Discovery D670	^99m^Tc-MDP
Lin et al. [39]	2023	R	LC	115/252	834 ± 41	N/A	N/A	6/15	Yes	Flash 3D (4/8)	Siemens Symbia Intevo	^99m^Tc-MDP

N/A—not available; PC—prostate cancer; VC—various cancer; BC—breast cancer; LC—lung cancer; R—retrospective; and P—prospective.

**Table 4:** Diagnostic accuracy of quantitative SPECT-CT in bone lesions.

Author	Metastatic and Benign Lesions SUVs (g/mL)			Cut-Off Value (g/mL)	Area under the Curve (AUC)	Sensitivity (%)	Specificity (%)
	ML/BL	ML SUVmax	BL SUVmax				
Kuji et al. [34]	126/114	40.9 ± 33.5	16.7 ± 6.7	N/A	0.924	N/A	N/A
Tabotta et al. [35]	265/24	35.0 ± 24.6	14.2 ± 3.8	19.5	0.947	87	92
Rohani et al. [36]	122/89	36.6± 24.8	12.6 ± 9.0	20.0	0.874	74	85
Zhang et al. [31]	48/40	24.8 ± 16.3	15.9 ± 8.5	17.7	0.687	N/A	N/A
Gherghe et al. [37]	236/179	32.6 ± 16.4	10.2 ± 4.7	16.6	0.974	92	93
Ikeda et al. [38]	252/140	25.4 ± 15.7	6.99 ± 2.58	11.2	0.933	87	93
Lin et al. [39]	252/140	23.8 ± 14.3	9.7 ± 7.4	11.1	0.909	88	81

ML—metastatic lesions and BL—benign lesions.

**Table 5:** Mean, standard deviation, and range for the SUVmax and cut-off value.

	Metastases SUVmax (g/mL)	Benign Lesions SUVmax (g/mL)	Cut-Off Value (g/mL)
Mean and standard deviation	32.2 ± 6.7	13.2 ± 2.9	17.0 ± 3.4
Range	23.8–40.9	9.7–16.7	11.1–20.0

**Table 6:** General data of SPECT-CT follow-up studies.

Authors	Year of Publication	Study Design	Number of Patients	Cancer Type	Metastatic Lesions (n)	Injected Activity (MBq)		Injection-Scan Time (min)		Interval between Scans (Months)	Scanner Type
						TP1	TP2	TP1	TP2		
Beck et al. [11]	2016	R	19	PC/BC	52	573.2 ± 105.4	542 ± 80.4	231	234	3–24	Siemens Symbia Intevo
Gherghe et al. [14]	2023	P	75	BC	249	659.7 ± 105.0	665.6 ± 96.4	181	182	6–8	GE Discovery 670DR

R—retrospective; P—prospective; PC—prostate cancer; BC—breast cancer; and TP—time point.

**Table 7:** Aims and statistical data of SPECT-CT follow-up studies.

Authors	Aim of the Study	Statistical Analysis of the Data		
		Qualitative Whole-Body Scintigraphy (WBS)	Qualitative SPECT/CT	Quantitative SPECT/CT
Beck et al. [11]	Assess inter and intra-observer agreement in qualitative vs. quantitative SPECT-CT follow-up by performing Cohen Kappa statistics (k)	k = 0.46 (moderate inter-observer agreement)	k = 0.35 (fair inter-observer agreement)	k = 0.94 (almost perfect inter-observer agreement)
Gherghe et al. [14]	Assess the correlation between qualitative and quantitative image interpretation methods in SPECT-CT follow-up by performing Spearman rho statistics (r)	r = 0.608 (moderate correlation between WBS and quantitative SPECT-CT)	r = 0.711 (strong correlation between qualitative and quantitative SPECT-CT)	Not applicable

r—correlation coefficient and k—inter-observer agreement coefficient.

## Data Availability

All data generated or analyzed during this study are included in the manuscript.
